# Relationship among perceived stress, xerostomia, and salivary flow rate in patients visiting a saliva clinic

**DOI:** 10.1007/s00784-018-2393-2

**Published:** 2018-03-09

**Authors:** Marjolein S. Bulthuis, Derk H. Jan Jager, Henk S. Brand

**Affiliations:** 1 Department of Oral Biochemistry, Academic Center for Dentistry Amsterdam (ACTA), Amsterdam Movement Sciences, University of Amsterdam and Vrije Universiteit Amsterdam, Gustav Mahlerlaan 3004, 1081 LA Amsterdam, the Netherlands; 2 Department of Oral and Maxillofacial Surgery and Oral Pathology, VU University Medical Center, Amsterdam Movement Sciences, Amsterdam, the Netherlands; 3 Center for Special Care Dentistry (Stichting Bijzondere Tandheelkunde), Amsterdam, the Netherlands; 4 Department of Oral Health Sciences, KU Leuven and University Hospitals Leuven, Leuven, Belgium

**Keywords:** Perceived stress, Xerostomia, Saliva, OHIP

## Abstract

**Objective:**

This aimed to assess the potential role of chronic stress in saliva secretion, xerostomia, and oral health in a population attending a saliva clinic.

**Materials and methods:**

Data of 114 patients who met the inclusion criteria and completed all questionnaires were analyzed in this study. Participants completed several validated questionnaires, including the Perceived Stress Scale, the Oral Health Impact Profile (OHIP-14), Xerostomia Inventory (XI), and Bother xerostomia Index (BI). Subsequently, the unstimulated, chewing-stimulated, and citric acid-stimulated saliva secretion rates were determined gravimetrically. Data were evaluated using Spearman’s correlation analysis and the Mann–Whitney U test.

**Results:**

A significant correlation was observed between perceived stress and XI score (*r* = 0.312, *p* = 0.001), as well as between perceived stress and BI score (*r* = 0.334, p = 0.001). Stress levels also were significantly associated with OHIP-14 scores (*r* = 0.420, *p* < 0.001), but an association between experienced stress and salivary flow rate could not be established.

**Conclusion:**

In this population, perceived chronic stress seems to be related to several aspects of dry mouth, including the perception of dry mouth, suffering from dry mouth, and the impact on quality of life. These effects were independent of the use of psychotropic medication. No actual reduction in salivary flow was found. Further studies to explore the causal linkage of stress with xerostomia seem warranted.

**Clinical relevance:**

Perceived chronic stress seems to be related with several aspects of dry mouth. This finding might be relevant in future prevention and treatment of xerostomia.

## Introduction

Saliva is a versatile and essential fluid that lubricates and protects the oral cavity and makes it possible to taste, swallow, and speak [1]. A reduced salivary flow imposes several risks to the oral cavity, such as an increased susceptibility to caries, tooth demineralization, fungal infections, and mucosal lesions. Therefore, a reduced salivary flow may contribute to a reduced quality of life [2].

Hyposalivation can be defined as the objective measurement of reduced salivary secretion while xerostomia is the subjective feeling of a dry mouth that the patient experiences [3]. The prevalence of xerostomia in the general population is high: 13–26% for men and 20–46% for women [4]. Head and neck irradiation, autoimmune diseases such as Sjögren’s syndrome, and use of xerogenic medication are main causes of a reduction in salivary flow rate [5, 6].

Results of some studies have indicated that different emotions can decrease or enhance salivary flow [7]. In addition, psychological disorders, like depression and anxiety, may be associated with xerostomia [8]. Anxiety and fear can potentially influence saliva secretion through pathways in the amygdala, hypothalamus, and brainstem [9].

The relationship between perceived stress and hyposalivation or xerostomia is addressed in two types of studies: those evaluating the role of acute stress in saliva secretion, and those that discuss the relation between chronic stress and saliva secretion or xerostomia. The relation between acute stress and salivary flow has frequently been studied and seems to depend on the type of stressor, the study design, and the population studied. Salivary flow rate is reduced by stressors such as taking academic exams [10, 11] or completing a memory test [12]. In contrast, watching a surgical video [12], performing computer tasks [13], or exposure to the Trier Social Stress Test [14] can enhance salivary flow. Winners of an international judo competition reported higher levels of cognitive anxiety and showed higher levels of salivary flow in comparison with losers [15]. Other studies could not establish changes in salivary flow caused by experimental stressors like presenting a public talk [16] or watching a surgical video [17]. The increase or decrease in salivary flow possibly depends on the personality of the subjects [18]. It has been suggested as well that an active coping stressor will decrease salivary flow while a passive coping stressor will enhance it [12].

Chronic stress may cause different changes in saliva secretion in comparison with acute stress [19]. Evidence regarding the relation of chronic stress with salivary secretion is scarcer. Studies have not identified a relation between self-reported measures of stress with a reduced stimulated or unstimulated salivary flow [20] even though stress [21], anxiety [8, 21], and xerostomia seem to be related.

The aim of this cross-sectional study was to assess the potential role of chronic stress in saliva secretion, xerostomia, and oral health in a population visiting a saliva clinic.

## Material and methods

### Study design

To address the research purpose, the investigators designed and implemented a single-center cross-sectional study. Samples and questionnaires were collected though convenience sampling from 177 patients who attended the saliva clinic of the Centre for Special Care Dentistry (Stichting Bijzondere Tandheelkunde, Amsterdam, the Netherlands) between December 2011 and December 2015. Patients were referred to the saliva clinic by dentists, physicians, or medical specialists. Patients were included in this study if they completed the analyzed questionnaires and excluded if they were using psychoanaleptics or psycholeptics [22] at the time of the examination or had a history of chemotherapy or radiation therapy in the head/neck region.

Reporting of this study conforms to the STROBE statement [23].

### Data collection methods

Case report forms (CRFs) were designed to collect data in a standardized manner. One data abstractor with specialized knowledge of the research question (MB) performed data abstraction from the medical charts to the CRFs to prevent incorrect transfer of data from the medical record. In addition, random checks were performed prior to data entry according to the 100–20 rule, in which 100% of the data is checked in 20% of the CRFs and 20% of the most important data was checked in 100% of the CRFs to prevent mistakes in data retrieval [24].

### Variables

Participants were invited to complete several validated questionnaires before the examination. To determine the perceived stress in the last month, a Dutch translation of the shortened version of the Perceived Stress Scale (PSS) was used [25]. This questionnaire consists of 10 items on a five-point scale. The degree to which the patient is affected by dry mouth was determined by the Bother xerostomia Index (BI) [26]. This index consists of a single score from 0 to 10, given by the patient. The Dutch translation of the Oral Health Impact Profile (OHIP-14) was used to measure oral health-related quality of life [27]. The summed score of the Dutch version of the OHIP-14 varies between 14 and 70. The symptoms of xerostomia were measured by the Xerostomia Inventory (XI) [3]. This multi-item method includes a wide range of xerostomia symptoms scored on a five-point scale, resulting in a score ranging from 11 (no xerostomia) to 55 (most severe xerostomia possible). The subjects were extra- and intraorally examined by a clinician, and the medical history was determined [28].

Unstimulated whole saliva, chewing-stimulated whole saliva, and citric acid-stimulated saliva were collected in a standardized manner. Patients were instructed to refrain from eating, drinking, chewing gum, brushing teeth, using mouthwash, and smoking for 60 min prior to visiting the clinic. All assessments were made between 8:00 a.m. and 12:00 p.m. to minimize fluctuations associated with the circadian rhythm of salivary secretion [29].

At the time of the visit, each patient was placed in a quiet room and asked to sit in an upright position. Unstimulated saliva was collected by the draining method in a pre-weighed plastic container [30]. Patients were instructed to begin collecting saliva immediately after an initial swallow and to expectorate into the container as soon as saliva had accumulated. During the collection period (5 min), patients were not allowed to swallow. Stimulated saliva was collected by chewing on a piece of Parafilm (5 × 5 cm, Parafilm M, Pechiney Plastic Packaging Company, Chicago, IL, USA). For collection of citric acid-stimulated saliva, the tongue of the patient was swiped every 30 s with a cotton roll soaked in 4% citric acid (pH 2), and saliva was collected for 2 min in a third container. Dentures were allowed to be worn during saliva collection. After the collection period, the plastic containers were reweighed, and the collected volume was determined by subtracting the weight of the container prior to collection. Salivary flow was calculated by dividing the collected volume (1 g of saliva = 1 mL) by collection time (min), and values are expressed in mL/min [30].

### Data analysis

Ordinal and continuous parameters are both presented as median, because all continuous data were not normally distributed (Shapiro–Wilk test: *p* < 0.01). The spread is presented as interquartile range (IQR), noted as 25th and 75th percentile. Differences between the participants who experienced relatively high stress and the group that experienced relatively low stress were examined using the Mann–Whitney U test.

Possible associations between experienced stress and salivary flow, XI score, BI score, and OHIP-14 score were explored with a bootstrapped Spearman rank correlation test (1000× bootstrapping). Data were analyzed using SPSS, version 23.0 (IBM Corp, Armonk, NY, USA). A significance level (α) of 0.05 was chosen for all tests.

## Results

Of the 177 patients who visited the saliva clinic, 42 were excluded because of the use of psychotropic medication, 5 because they had undergone radiation therapy in the head/neck region, and 16 because they did not complete all items of the PSS. This resulted in a study population of 114 patients, of which characteristics are summarized in Table 1.

**Table 1:** Population characteristics

Variable	*n* = 144
Age in years, mean (range)	50.8 (12–99)
Female gender, *n* (%)	63 (55%)
Medication use, *n* (%)	59 (52%)
Number of medications, median (range)	1 (0–12)
Removable prosthesis, *n* (%)	23 (20%)
Main reason for visiting the saliva clinic, *n* (%)	
Xerostomia	51 (45%)
Tooth wear or caries	49 (43%)
Hypersalivation	4 (4%)
Intra-oral pain	4 (4%)
Miscellaneous reasons	3 (3%)

Of this study population, salivary flow rates and XI score were available for all patients, the BI score was available for 110 patients, and 104 patients completed the OHIP-14 questionnaire.

The median unstimulated salivary secretion rate in this population was 0.18 mL/min (IQR 0.08–0.33), the median chewing-stimulated secretion rate was 0.81 mL/min (IQR 0.38–1.33), and the median acid-stimulated secretion rate was 1.95 mL/min (IQR 1.05–2.86)*.* Forty-one patients had an unstimulated salivary flow rate less than 0.1 mL/min. Causes of this hyposalivation were Sjögren’s syndrome (*n* = 18; according to AECG criteria [31]), medication-induced hyposalivation (*n* = 4), or miscellaneous or not specified (*n* = 19).

**Fig. 1:**
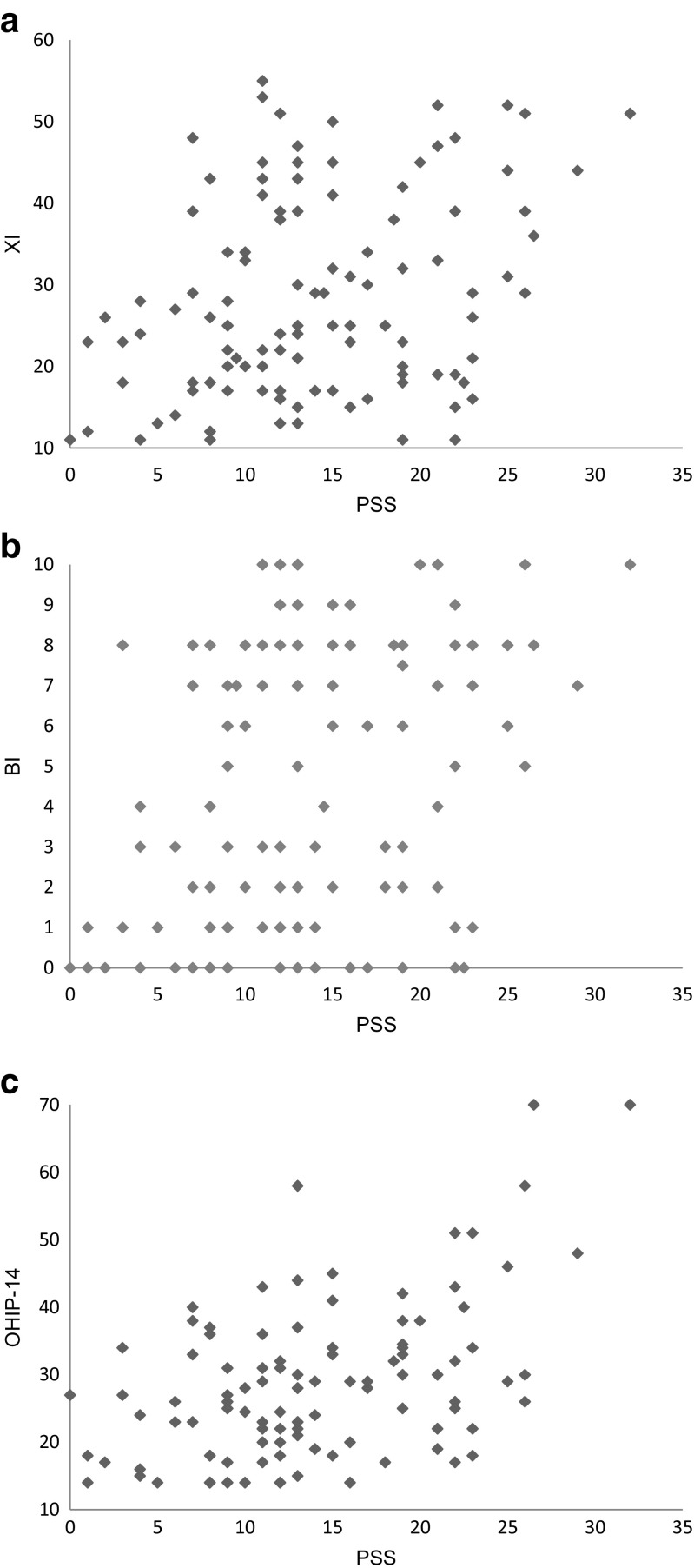
Correlations between Perceived Stress Scale and **a** XI score, **b** BI score, and **c** OHIP-14 score

The PSS ranged between 0 (no stress) and 40 (extremely high experienced stress), with a median value in the study population of 13 (IQR 9–19). The bootstrapped correlations between PSS score and secretion rate, XI score, BI score, and OHIP-14 score are presented in Table 2. The statistically significant correlations are shown in Fig. 1a–c.

**Table 2:** Correlation of Perceived Stress Scale with other variables

Variable	*r*	*p* value
Saliva secretion rate		
Unstimulated	− 0.157	0.117
Chewing stimulated	− 0.103	0.306
Acid stimulated	− 0.195	0.051
Xerostomia Inventory	0.312	0.001*
Bother xerostomia Index	0.334	0.001*
Oral Health Impact Profile	0.420	< 0.001*

*r* Spearman’s rho correlation coefficient

*Correlation is significant at the 0.01 level (two-tailed)

Participants who reported relatively high stress levels (PSS ≥ 13) were compared with those who reported relatively low stress (PSS < 13). Those who experienced high stress had significantly higher BI scores (Mdn = 6, IQR 2–8 vs Mdn = 2.5, IQR 1–7; *p* = 0.017) and XI scores (Mdn = 29, IQR 19–41 vs Mdn = 23, IQR 17–34; *p* = 0.041) than participants with low stress. The scores for the different items of the OHIP-14 questionnaire, as well as the summed score of the 14 items, are presented in Table 3. Participants who experienced high stress levels had lower unstimulated flow rates (Mdn = 0.18 mL/min, IQR 0.06–0.28) in comparison with those with low stress levels (Mdn = 0.22 mL/min, IQR 0.08–0.36), but this difference did not reach statistical significance.

**Table 3:** Comparison between high and low experienced stress and median scores and IQR of different items of the OHIP-14

OHIP-14 item^a^	Low stress, median (*n* = 48)	IQR	High stress median (*n* = 56)	IQR	*p* value
Functional limitation					
Trouble pronouncing words	1	1–2	1	1–3	0.043
Sense of taste worse	1	1–2	2	1–3	0.046
Physical pain					
Painful aching in mouth	2	1–3	3	2–3	0.361
Uncomfortable to eat	2	1–3	3	1–3	0.189
Psychological discomfort					
Self-conscious	1.5	1–3	3	1.3–3.8	0.018
Felt tense	1	1–3	3	2–3	0.006
Physical disability					
Unsatisfactory diet	1	1–2	2	1–3	0.003
Had to interrupt meals	1	1–2	1.5	1–2.8	0.028
Psychological disability					
Difficulty to relax	1	1–2	2	1–3	0.003
Embarrassed	2	1–3	3	2–4	0.008
Social disability					
Irritability with others	1	1–2	2	1–3	0.002
Difficulty doing usual jobs	1	1–1	2	1–3	0.001
Handicap					
Felt life less satisfying	1	1–2.8	3	1.3–3.8	0.001
Totally unable to function	1	1–1	1	1–2	0.027
Total OHIP-14 score	23	17–30.5	30	23–39.5	<0.001

^a^The score for each item ranges from 1 to 5

## Discussion

The study population consisted of patients who visited a saliva clinic because of complaints about salivary flow, xerostomia, or expected changes in salivary function. The unstimulated salivary flow in the study population was low (0.18 mL/min, IQR 0.08–0.33) in comparison with that of the general population (0.3–0.4 mL/min) [1].

In the present study, a moderate association was observed between perceived stress and xerostomia. These findings corroborate previous studies. Bergdahl et al. studied the relation between psychological factors and xerostomia in a randomly selected, non-hospitalized population [21]. The authors concluded that psychological factors such as depression, anxiety, and stress play an important role in causing xerostomia. Veerabhadrappa et al. [8] investigated the prevalence of xerostomia in different psychological disorders. Xerostomia was reported in 51% of patients suffering from anxiety and 27% of controls. A positive association was established between psychological alterations and xerostomia and visible dryness of oral mucosa and lips as well.

Anxiety and fear may potentially affect salivary secretion through pathways in the amygdala, the hypothalamus, and the brainstem [9]. Although a lower unstimulated salivary flow rate was found in the high stress group, this difference did not reach statistical significance. Hugo et al. [20] also found no relation between self-reported stress and salivary flow in a population aged 50 years and older. Even though they concluded that being a dementia caregiver, which was assumed to be a proxy for chronic stress, was a risk indicator for low stimulated salivary flow.

In the present study, salivary parameters are only measured when patients visit the saliva clinic. The lack of salivary data before onset of disease or complaints makes it impossible to establish a causal relationship between experienced stress and salivary flow rate.

Participants who used psychotropic medication at the time of the examination or had undergone radiation therapy in the head/neck region were excluded from this study because these factors could have influenced both the experienced stress [32, 33] and the salivary flow [5, 6]. Nevertheless, even after exclusion of these patients, the study population was rather heterogeneous and included patients diagnosed with diabetes, Sjögren’s syndrome, and patients who used medication with xerogenic potential. These conditions could have influenced salivary flow as well [34] and could potentially have acted as confounders.

The OHIP-14 questionnaire was used in the present study to measure the influence of oral health on quality of life. People who experienced relatively high levels of stress had higher scores on most items of the OHIP questionnaire, as well as a higher summed score of the 14 items. The negative relation between experienced stress and oral health-related quality of life, measured with the OHIP, or other questionnaires, is confirmed in different populations. Thomson et al. [35] concluded that there is an association between a negative emotionality, which includes the stress reaction, and OHIP scores in a birth cohort in New Zeeland. Acharya et al. [36] concluded that work stress may be an important predictor for a poor oral health-related quality of life in information technology professionals in south India.

The results of the present study raise the question whether lowering stress levels could influence xerostomia symptoms and whether stimulating salivary flow could affect the perceived stress level. Conflicting results have been reported about the changes in salivary flow rate after removal of an acute stressor [12]. For example, salivary flow of patients exposed to an unpleasant dental treatment, such as an endodontic treatment, is reduced. When the subjective anxiety is reduced, though, salivary flow increases to normal levels [37]. On the other hand, Borgeat et al. [38] could not find a difference in salivary flow after a stressful task in comparison with relaxation.

Few studies have examined changes in saliva after lowering chronic stress. Naumova et al. [39] concluded that patients with dental phobia had lower salivary secretion rates than controls before oral examination. A psychotherapeutic treatment that diminished the anxiety state led to equalization of the secretion rates in both groups. Cho et al. [40] concluded that the salivary secretion rate, OHIP scores, and xerostomia were positively influenced by an oral health promotion program for elderly women.

## Conclusion

We can conclude that in patients visiting a saliva clinic, perceived chronic stress seems to be associated with several aspects of dry mouth, including the perception of dry mouth, suffering from dry mouth, and its impact on the quality of life. These effects were independent of the use of psychotropic medication. No actual relation between perceived stress and salivary flow could be established. Further studies are warranted to explore the causal linkage of stress with xerostomia.
